# Floral Organogenesis in Three Members of the Tribe Delphinieae (Ranunculaceae)

**DOI:** 10.3390/plants8110493

**Published:** 2019-11-11

**Authors:** Hongli Chang, Stephen R. Downie, Hongli Peng, Fengjie Sun

**Affiliations:** 1 Shaanxi Key Laboratory for Animal Conservation, School of Life Sciences, Northwest University, Xi’an 710069, China; 2 Department of Plant Biology, University of Illinois at Urbana-Champaign, Urbana, IL 61801, USA; sdownie@illinois.edu; 3 Hebei University of Environmental Engineering, Qinhuangdao 066102, China; penghongli@hebuee.edu.cn; 4 School of Science and Technology, Georgia Gwinnett College, Lawrenceville, GA 30043, USA

**Keywords:** Delphinieae, *Aconitum*, *Consolida*, *Delphinium*, monosymmetry, floral organogenesis, Ranunculaceae

## Abstract

Three species (*Aconitum taipeicum*, *Delphinium giraldii*, and *Consolida ajacis*) of the tribe Delphinieae (Ranunculaceae) were examined using scanning electron microscopy and histological methods. The results showed that members of Delphinieae differ from their polysymmetrical relatives by four unique features: (1) a spiral phyllotaxis of their perianth and stamens, and a series of carpels, which initiated superficially in a whorl-liked arrangement; (2) sepal 2 being the largest one among the five sepals and becoming helmet-shaped or having a spur; (3) petals 2 and 5 initiated adaxially of sepal 2 and also becoming spurred; and (4) the monosymmetry of the first flower becoming established when sepal 2 becomes the largest. Major differences among the species include the timing of development of the second series; the fusion of two petals into a single one in *C. ajacis*; and, during early developmental stages, the two young spurred petals giving rise to a stalk and two bulges in *A. taipeicum*, a single bulge in *D. giraldii*, or an arch blade in *C. ajacis*. The unequal growth of the perianth, together with the reduction and the rearrangement of the carpels, are critical in inducing the symmetrical transformation of the flowers.

## 1. Introduction

The Ranunculaceae tribe Delphinieae Warming is characterized by distinctive flowers possessing strong zygomorphy. The second series (“series” defined as a set of organs of the same kind regularly surrounding the floral center) [1,2] of floral organs is morphologically differentiated and diverse, with elaborate petaloid, spurs, or reduced. The spurred petals in the second series are termed variously as petals [2,3,4,5], nectar-leaves [6], staminodia [7], or nectary organs [8]. The second series is thought to have originated from either stamen-like organs (andropetaloidy) [4,9,10,11,12,13] or bract- or leaf-like organs (bracteopetaloidy) [14,15]. However, molecular evidence showed that regardless of the morphological origins, the formation of petals may be specifically regulated at the molecular level [2]. The various shapes and sizes of this second series of floral organs determine their spatial arrangement, resulting in a flower with a unique monosymmetrical structure.

The tribe Delphinieae (*Aconitella* Spach, *Aconitum* L., *Consolida* (DC.) Gray, *Delphinium* L., *Gymnaconitum* (Stapf) Wei Wang and Z.D. Chen, and *Staphisagria* J. Hill) comprises approximately 650 to 700 species, representing about a quarter of all species of Ranunculaceae [11,15,16,17,18]. The tribe’s center of distribution is located in southwest China and the eastern Himalayas, where *Aconitum* has 166 species [19] and *Delphinium* has some 150 species [20]. Flowers of other members of Ranunculaceae have the polysymmetrical pattern, suggesting that flowers with a monosymmetrical structure possess a developmental mechanism distinct from those that are polysymmetrical. Polysymmetry is considered ancestral in angiosperms [21], whereas zygomorphy has evolved multiple times independently [22,23,24]. Therefore, the monosymmetrical structure seen in Delphinieae is suggestive of an advanced feature in Ranunculaceae [5,15,18,22,25]. With the presence of spurred petals, more or less reduced petals, and laminar petals in an individual flower, the tribe Delphinieae provides a model system in which to investigate when and how the monosymmetrical pattern initiates [5].

Genera *Aconitum*, *Delphinium,* and *Consolida* are classified in either subfamilies Ranunculoideae [26,27] or Helleboroideae based on results of phylogenetic analyses of molecular and morphological data [25,28,29,30]. The relationships among these three genera are either unresolved [7,26], consider *Consolida* and *Delphinium* as sister taxa [29,30], or suggest that *Consolida* is embedded within *Delphinium* [16,31,32]. Floral developmental traits contain evolutionary information and can be used to infer phylogenetic relationships, and such ontogenetic features have been used previously to investigate phylogenetic relationships in Ranunculaceae, including *Caltha* L. and *Trollius* L. [33]; tribe Adonideae [34]; *Helleborus* L. and *Nigella* L. [35]; tribe Thalictroideae [36]; as well as in many other families, such as Tropaeolaceae [37], Fabaceae [38], and Leguminosae [39].

Previous studies have explored developmental mechanisms in Delphinieae by focusing on their perianth and primarily their spurred petals, because they are complex and the most visible parts of the flower [3,4,5,8,17,40,41,42,43,44,45]. However, a complete ontogenetic series of all floral organs and phylogenetic studies based on floral ontogenetic characters for the three species (*Aconitum taipeicum*, *Delphinium giraldii*, and *Consolida ajacis*) in Delphinieae are still lacking. To explore whether the other floral organs play a role in the formation of monosymmetry in Delphinieae, herein we re-examine the development and structure of its flowers. Ranunculaceae exhibit a stamen elongation sequence in accordance with the stamen maturation sequence, with the former being centripetal, centrifugal, or bidirectional [33,34,46,47,48]. In *Helleborus* (Ranunculaceae), the stamen elongation sequence is centrifugal, while the maturation sequence of the microspores is centripetal [35]. The unique developmental pattern of *Helleborus* prompted us to investigate the stamen development in Delphinieae in order to further explore the androecial variation in the family.

The objectives of this study are as follows: (1) to describe floral organogenesis in *Aconitum taipeicum*, *Delphinium giraldii*, and *Consolida ajacis*; (2) to identify unique floral features that distinguish tribe Delphinieae from other Ranunculaceae; (3) to understand the developmental mechanism controlling the floral transition towards a monosymmetrical structure; and (4) to describe the sequence of stamen elongation and maturation in Delphinieae. The selection of these three species is based on the fact that a complete ontogenetic series of all floral organs in these plants is still lacking. The floral organogenesis in these plants could shed insight on the formation of monosymmetry in Delphinieae.

## 2. Results

### 2.1. Aconitum Taipeicum

#### 2.1.1. Flowers at Anthesis

**Figure 1:**
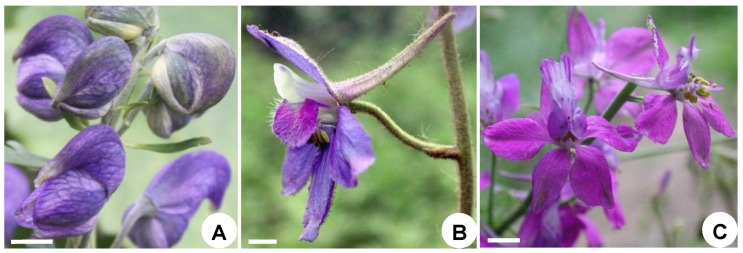
Mature flowers of Delphinieae. (**A**), *Aconitum taipeicum*; (**B**), *Delphinium giraldii*; (**C**), *Consolida ajacis.* Scale bar = 0.4 cm.

There are two linear bracteoles at the middle of the pedicel, the flower is bisexual and monosymmetrical, and the five sepals are blue and petaloid. The uppermost sepal forms a distinct helmet-shaped hood (galea) (Figure 1A). There are two petals, each with a hollow spur at its apex, and these are located at the upper side of the flower. The spurs are concealed within the hood. There are six reduced petals and 30–40 stamens, the latter with a tubercle on the base of each side of the filament. There are five distinct carpels. All floral elements are free.

#### 2.1.2. Organ Initiation

**Figure 2:**
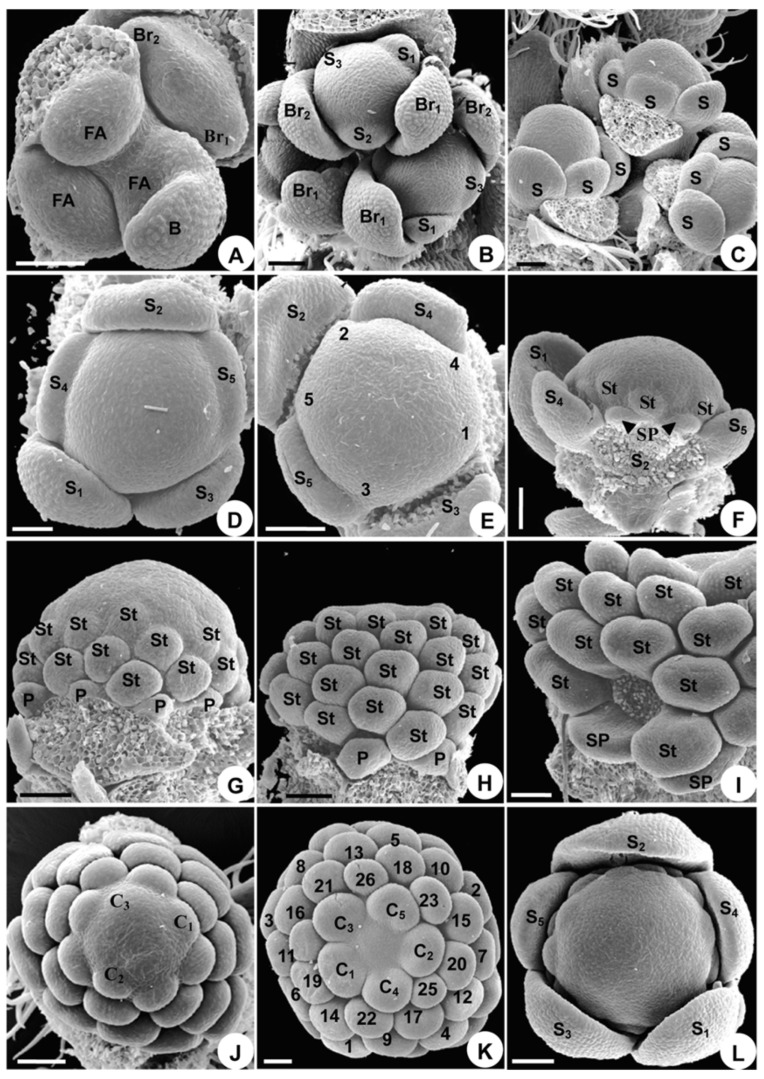
Floral organogenesis in *Aconitum taipeicum*. (**A**), floral apex arises from the axils of bract and two bracteoles. (**B**), initiation of sepals from sepals 1 to 3 (subscript), showing sepal 2 situated on the adaxial side of the inflorescence. (**C**), side view of sepals, showing their crescent-shape, from initially truncate to finally round. (**D**), the outer pentamerous whorl of the flower when five sepals are initiating. (**E**), five hemispherical petal primordia initiate in a spiral sequence. (**F**), side view of primordia 2 and 5, which will develop into spurred petals, and the initiation of stamen primordia, showing similar shapes and sizes of petal and stamen primordia. (**G**–**I**), developmental stages of petals and stamens showing the parastichies that consist of petals and stamens. (**G**), delayed growth of petals in comparison with stamens. (**H**), an older stage than that shown in G, showing the petals are still smaller than the stamens. (**I**), the spurred petals begin to enlarge. (**J**), carpel primordia arise. (**K**), five carpels arranged in a whorl and stamens at their initiation order. (**L**), sepal 2 is the largest in size among all sepals, making the flower slightly monosymmetrical in shape. B, bract; Br, bracteole; C, carpel; FA, floral apex; P, petal; S, sepal; SP, spurred petal; St, stamen. Numbers indicate the initiation sequence. Scale bars: (**A**–**L**), 100 µm.

Flowers arise as elliptical primordia in the axil of a subtending bract (Figure 2A). Two bracteoles subsequently initiate on each side of the flower bud (Figure 2A); these are crescent-shaped and become different in size shortly after inception (Figure 2B).

The first sepal arises abaxially and inserts itself towards the first bracteole (Figure 2B). Subsequently, the other four sepals initiate, following a spiral sequence (Figure 2B–D). The sepals are crescent-shaped and truncate (Figure 2B–D). After the sepals arise, the floral apex forms an outer pentamerous whorl (Figure 2D).

Subsequently, 35–45 hemispherical protuberances initiate from the periphery of the floral apex, following a spiral initiation sequence (Figure 2E–G). Judging from their position and number, eight of these primordia are petals or reduced petals. Primordia 2 and 5 are located on two sides of sepal 2 (Figure 2E). The identity of the petal, reduced petal, and stamen primordia is often a matter of conjecture, as they are similar in shape and size during their early developmental stages (Figure 2F). The petal, reduced petal, and stamen structures make up a series of parastichies in a side view (Figure 2G,H).

Five carpels initiate at the same spiral sequence, but more rapidly in time (Figure 2J), and no distinctive difference in primordium shape appears to be present during the transition from androecium to gynoecium (Figure 2J). The carpels soon form a pentamerous whorl and become equal in size (Figure 2K). The remainder of the center apex is empty (Figure 2K), which is later occupied by the growing carpels. At this stage, the flower has the full complement of floral organs.

#### 2.1.3. Organ Development

**Figure 3:**
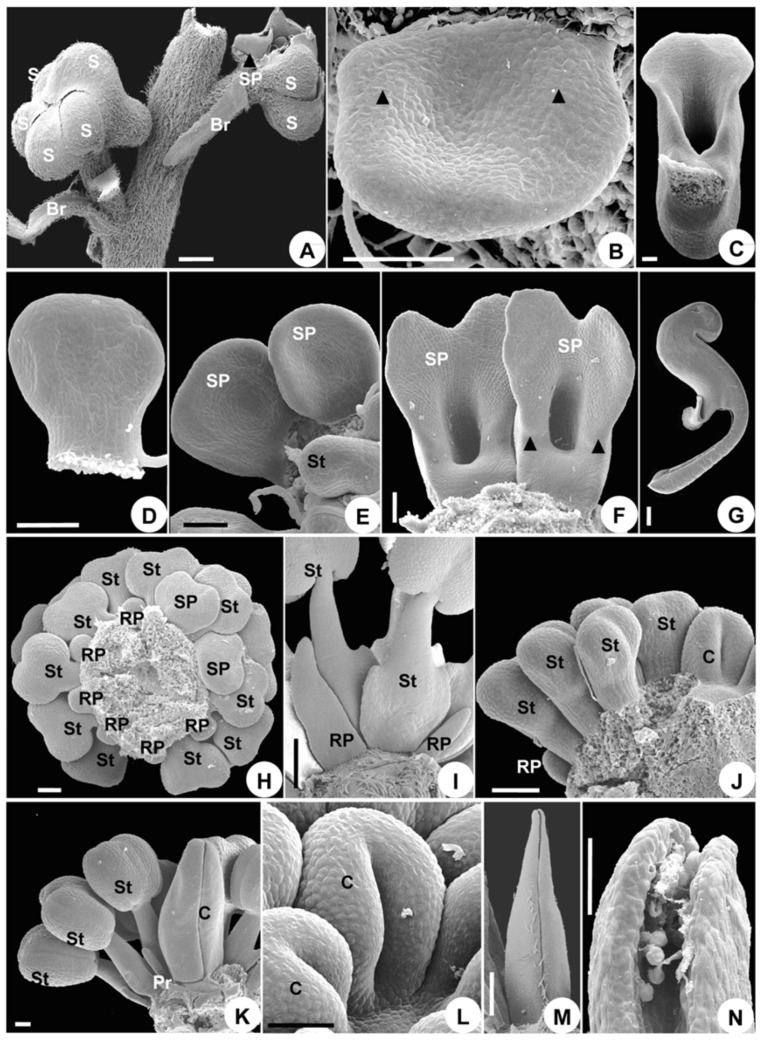
Floral organogenesis in *Aconitum taipeicum*. (**A**), the differentiation of sepals and the formation of symmetry. (**B**–**G**), petal development. (**B**), a middle depression and two bulges (arrows) at the edge of the petal. (**C**), the margin of the spurred petal grows upwards to form a ring along the two bulges. (**D**), petal grows into a blade with stalk. (**E**), the depression becomes evident. (**F**), the depression and two bulges (arrows) become more evident. (**G**), the petal develops into a labium, spur, and stalk. (**H**), bottom view of the spurred petals, reduced petals, and stamens, showing their different developmental stages. (**I**), the shapes of reduced petals prior to anthesis and the protuberances at the base of the staminal filament. (**J**), centripetal development of stamens and a cleft appear on the carpel. (**K**), carpel closure. (**L**), the horseshoe-shaped carpel. (**M**), carpel differentiating into ovary, style, and stigma. (**N**), the morphology of the stigma at anthesis, with the stigmatic surface covered with pollen grains. Br, bracteole; C, carpel; RP, reduced petals; Pr, staminal protuberances; S, sepal; SP, spurred petal; St, stamen. Numbers indicate the initiation sequence. Scale bars: (**A**–**F**,**H**,**J**–**L**,**N**), 100 µm; (**G**,**I**,**M**), 500 µm.

The sepals enlarge and enclose the other floral organs in a typical quincuncial pattern (Figure 2L). Sepal 2, located on the adaxial side, grows larger than the other sepals, making the flower slightly monosymmetrical in shape (Figure 2L). Sepal 2 then becomes helmet-shaped, sepals 4 and 5 become subrotund, and sepals 1 and 3 become oblong (Figure 3A). Thus, monosymmetry is first established by sepal enlargement (Figure 3A). 

The eight petals cease growing immediately after their initiation and are much smaller than the stamens (Figure 2G,H). Later, the spurred petals start to develop and then their sizes are somewhat similar to that of the stamens (Figure 2I), but the reduced petals remain rudimentary. Initially, the spurred petals enlarge and flatten (Figure 2I). A depression and two bulges appear in the middle of the round disk of the blade growing entirely from the upper side of the primordium, indicating the initiation of the spur (Figure 3B). The depression later becomes evident and continues to stretch down to form the spur (Figure 3C–G), while the basal part of the blade narrows and develops into a stalk (Figure 3D). The two bulges become more evident and the abaxial side of the petal grows into a bilobate margin (Figure 3F). This two-lobed margin grows upward and fuses into a ring along the two bulges (Figure 3C). The abaxial side of the ring is wider than its adaxial side (Figure 3C). The growth of the abaxial side of the ring is slower than that of the adaxial side, causing the ring to divide into two parts, each of a different shape and size (Figure 3G). The upper part forms the labium of the petal, while the lower part connects to the short stalk and becomes the claw (Figure 3G). The outgrowth of the two spurred petals further increases the monosymmetry of the flower (Figure 1A).

Compared with the spurred petals and stamens, the six reduced petal primordia remain rudimentary, but their development is still discernible (Figure 3H). They change their shapes slightly from rounded to laminar structures with acuminate apices (Figure 3I). Both spurred and reduced petals are arranged in a series and present a monosymmetrical structure (Figure 3H).

In a longitudinal series of stamens, the elongation and differentiation sequences are centripetal (Figure 3J,K). Two protuberances emerge gradually at the base of the filaments (Figure 3I,K). The androecium is polysymmetrical and this symmetry is maintained during the developmental stages.

The expansion of the margin of the carpel primordia results in an elongated cleft from across the top to the bottom of the centripetal basal side (Figure 3J). Subsequently, the cleft heightens and appears horseshoe in shape (Figure 3L). The flanges on either side of the cleft then extend around to become appressed, closing the cleft (Figure 3K). Eventually, the cleft develops into a ventral suture (Figure 3M). The middle part of the carpel is inflated to form the ovary. The near top portion becomes curved towards the adaxial side of the flower, tapering gradually to become a narrow style (Figure 3M). The stigma at anthesis is terminal, non-papillate, bisected by the terminus of the cleft, and no wider than the style (Figure 3M,N). The gynoecium is polysymmetrical and maintains this symmetry during its developmental stages.

#### 2.1.4. Developmental Sequence of Microspores

**Figure 4:**
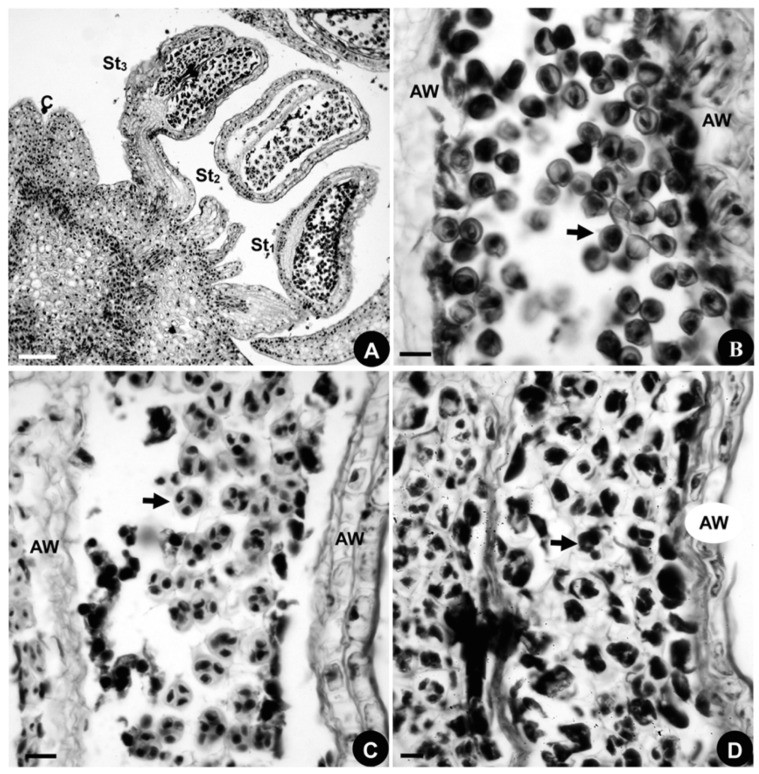
Microsporogenesis in *Aconitum taipeicum*. (**A**), a longitudinal section of a flower showing three stamens and the carpel. (**B**), the microspores (arrow) in the outermost stamen. (**C**), the microspores in the tetracyte stage (arrow) in the middle stamen. (**D**), the microspores in the microsporocyte stage (arrow) in the innermost stamen. AW, anther wall; C, carpel; St, stamen. Numbers indicate the initiation sequence. Scale bars: (**A**–**D**), 100 μm.

The longitudinal series consists of three stamens. Histological observations (Figure 4A) reveal that the microsporogenesis of the outermost stamen is in the microspore stage (Figure 4B), the middle stamen is in the tetracyte stage (Figure 4C), and the innermost one is in the microsporocyte stage (Figure 4D). Stamen maturation is in a centripetal direction, consistent with the stamen initiation and differentiation sequences. 

A comparison of floral organogenesis in five species of tribe Delphinieae, including the three species examined in our study and two with sufficient organogenesis characters, and *Nigella* is presented in Table 1.

### 2.2. Delphinium Giraldii

#### 2.2.1. Flowers at Anthesis

Two linear bracteoles occur in the middle of the pedicel, the flower is bisexual and monosymmetrical, and the five sepals are purple-blue in color and petaloid. The uppermost sepal has a backward pointing spur (Figure 1B). There are a total of four reduced petals that are situated on the adaxial side of the axis. The middle two petals, each placed inside the spurred sepal, have hollow spurs projecting into the sepal spur. The two bilateral petals are small, each with a claw and a bifid top. There are 18–30 stamens and 3 distinct carpels. All floral elements are free.

#### 2.2.2. Organ Initiation

**Figure 5:**
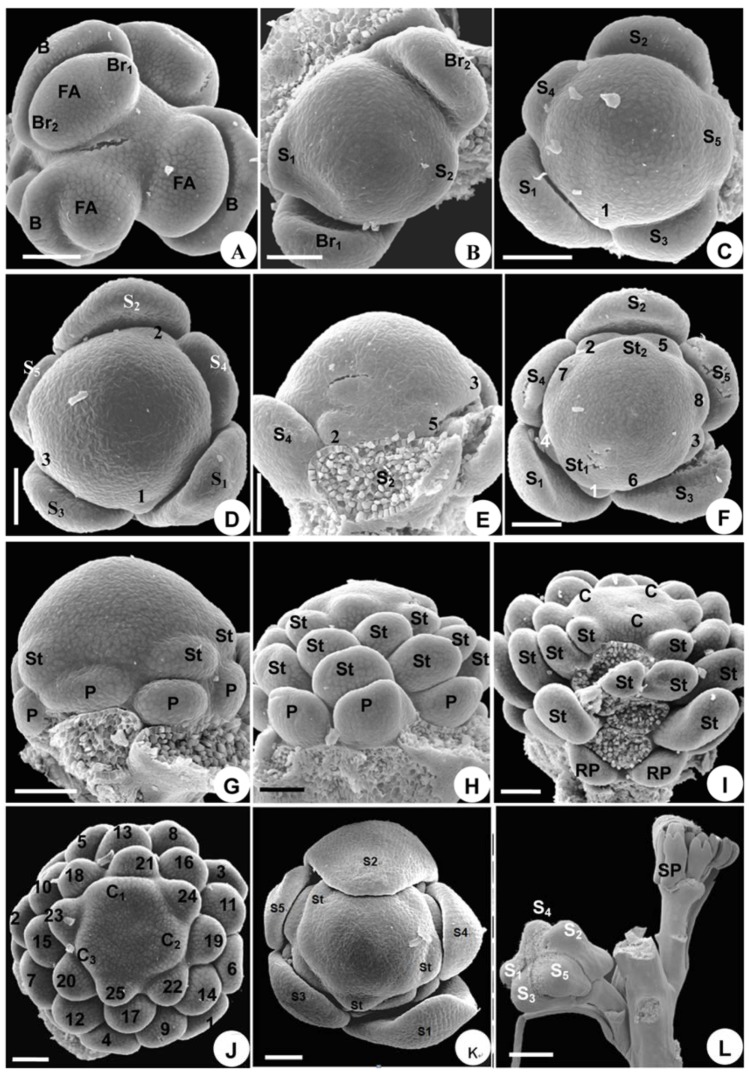
Floral organogenesis in *Delphinium giraldii*. (**A**), floral apex arises from the axils of bract and two bracteoles. (**B**), initiation of sepals 1 and 2. (**C**), initiation of sepals 1 to 5 and petal 1, showing their spiral order of initiation. (**D**), petals 1–3 arise in a spiral fashion as hemispherical protuberances. (**E**), two petals (2 and 5) are adaxial to sepal 2. (**F**), eight petals (1–8) and four stamen primordia arise, with initiation sequences indicated. (**G**), the more or less reduced petals are bigger than stamen primordia. (**H**), the parastichies and comparable growth of both petals and stamens. (**I**), the reduced petals cease to grow when the stamens begin to differentiate. (**J**), carpel primordia arise. (**K**), the early differentiation of sepals, showing the floral symmetry changing slightly from polysymmetry to monosymmetry. (**L**), the differentiation of sepals, showing the position of five sepals. B, bract; Br, bracteole; C, carpel; FA, floral apex; P, petal; RP, reduced petals; S, sepal; SP, spurred petal; St, stamen. Numbers indicate the initiation sequence. Scale bars: (**A**–**L**), 100 µm.

The initiation of the flower is nearly the same as that of *A. taipeicum,* with all of its organs initiated in a spiral sequence (Figure 5A–J). An apparent difference is that, in *D. giraldii*, the petal primordia are bigger than the stamen primordia (Figure 5G). The more or less reduced petals and stamens consist of a series of parastichies (Figure 5H). Three carpel primordia initiate rapidly to form a triangular whorl and become equal in size (Figure 5I,J).

#### 2.2.3. Organ Development

**Figure 6:**
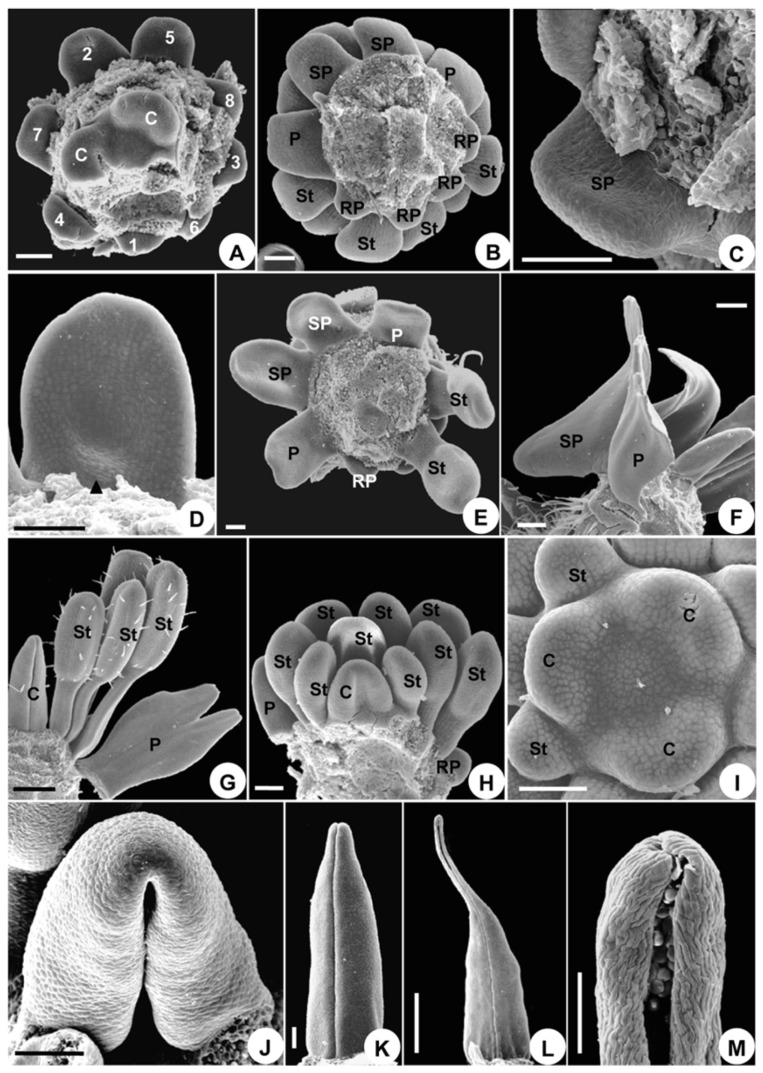
Floral organogenesis in *Delphinium giraldii*. (**A**), the differentiation of the second series, showing an unbalanced outline. (**B**), bottom view showing that the reduced petals have ceased growing. (**C**–**F**), petal development. (**C**), a spurred petal grows into a lamellar structure with an intact edge. (**D**), the petal grows into a bulge and a depression. (**E**), the evident depression on the spurred petal flanked by blade petals. (**F**), the spur elongates. (**G**), the shape of the blade petal and the centripetal development sequence of stamens are shown. (**H**), the shape of reduced petals, the centripetal developmental sequence of stamens, and the shape of carpel with a cleft developed are shown. (**I**–**M**), carpel development. (**I**), three carpels arranged in a whorl. (**J**), the cleft begins to fuse at the middle part of the carpel. (**K**), the cleft on the carpel closes. (**L**), the morphology of the carpel at anthesis. (**M**), the shape of the stigma at anthesis, with many pollen grains deposited upon it. C, carpel; P, blade petal; RP, reduced petals; SP, spurred petal; St, stamen. Numbers indicate the initiation sequence. Scale bars, **A**–**E**, **H**–**K**, **M**, 100 µm; **F**, **G**, 300 µm; **L**, 1 mm.

The growth of sepals is similar to that of *A. taipeicum* (Figure 5K,L). However, petal growth is different. The petals do not cease growing after their initiation, in contrast to the inner stamens (Figure 5G,H). After a while, only the reduced petals cease growing when the stamens begin to differentiate (Figure 5I and Figure 6B). The series then presents an unbalanced shape, showing a further transition between polysymmetry and monosymmetry (Figure 6A,B). Primordia 2, 5, 7, and 8 develop into petals (Figure 6A,B). Primordia 2 and 5, occurring on both sides of sepal 2, depress to form the spurred petals (Figure 6A,B). Primordia 7 and 8 form the laminar petals (Figure 6A,B), and primordia 1, 3, 4, and 6 develop into reduced petals (Figure 6A,B). The primordia of the spurred petals first enlarge to form a sessile elliptic blade (Figure 6C). Subsequent development continues from the central meristematic area, causing the region to show depression (Figure 6D). Simultaneously, the lower surface of the depression becomes thicker to form a bulge (Figure 6D,E). The depression elongates to form a spur (Figure 6F). Primordia 7 and 8 enlarge into a sessile blade petal (Figure 6E). This blade further elongates and differentiates into a short stalk and a bilobate blade (Figure 6G). The shape and size of the four abaxially reduced petals change slightly during floral development because of their rudimentary growth (Figure 6B,E,H).

The stamens develop centripetally (Figure 6G,H), just as they do in *A. taipeicum*. The development of the carpel is also nearly the same as that of *A. taipeicum* (Figure 6H–L). The stigma at anthesis is terminal, non-papillate, and bisected by the terminus of the cleft (Figure 6M).

#### 2.2.4. Developmental Sequence of Microspores

**Figure 7:**
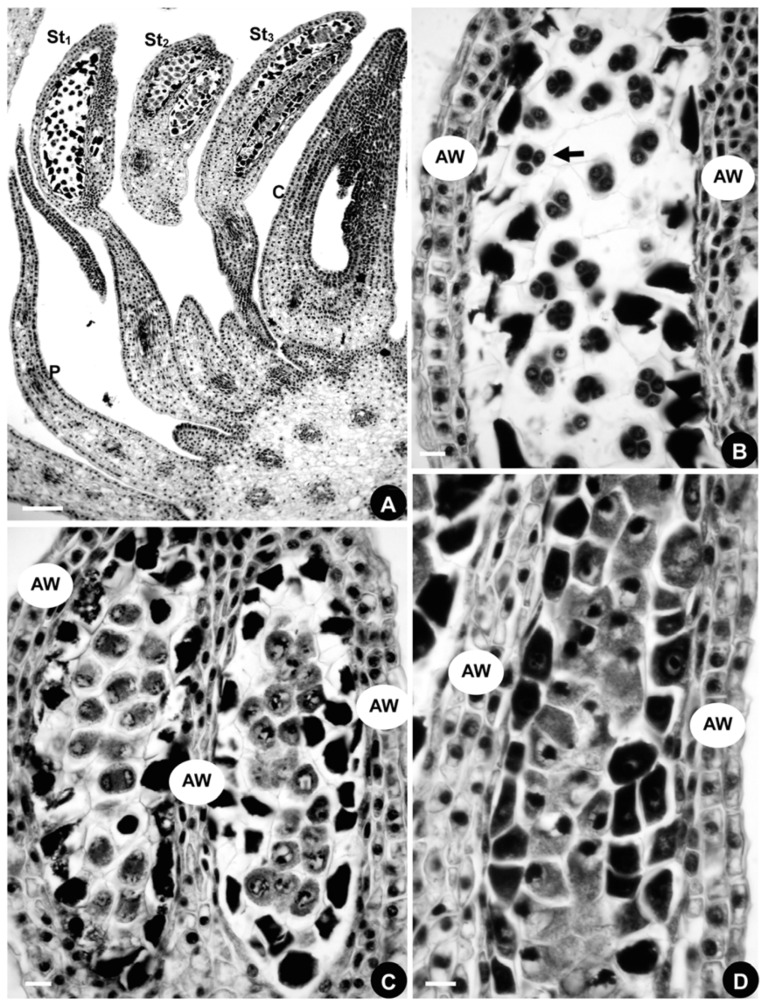
Microsporogenesis of *Delphinium giraldii*. (**A**), a longitudinal section of a flower showing three stamens. (**B**), the microspores are in the tetracyte stage (arrow) in the outermost stamen. (**C**), the microspores are in the diad stage in the middle stamen. (**D**), the innermost stamen contains the microspores in the microsporocyte stage. AW, anther wall; C, carpel; P, petal; St, stamen. Numbers indicate the initiation sequence. Scale bars: (**A**–**D**), 100 μm.

The developmental sequence of the microspores in *D. giraldii* is centripetal, just as it is in *A. taipeicum* (Figure 7A–D). A longitudinal section of a flower with three stamens is shown in Figure 7A. The histological observations show that the microspores are in the tetracyte stage in the outermost stamen (Figure 7B), the microspores are in the diad stage in the middle stamen (Figure 7C), and the innermost stamen contains the microspores in the microsporocyte stage (Figure 7D).

### 2.3. Consolida Ajacis

#### 2.3.1. Flowers at Anthesis

Two linear bracteoles occur in the middle of the pedicel, the flower is bisexual and monosymmetrical, and the five sepals are purple-colored and petaloid. The uppermost sepal has a long, backward-pointing spur (Figure 1C). One spurred petal is located on the upper side of the flower with a long spur projecting upward into the upper spurred sepal. There are 13–20 stamens and a solitary carpel.

#### 2.3.2. Organ Initiation

**Figure 8:**
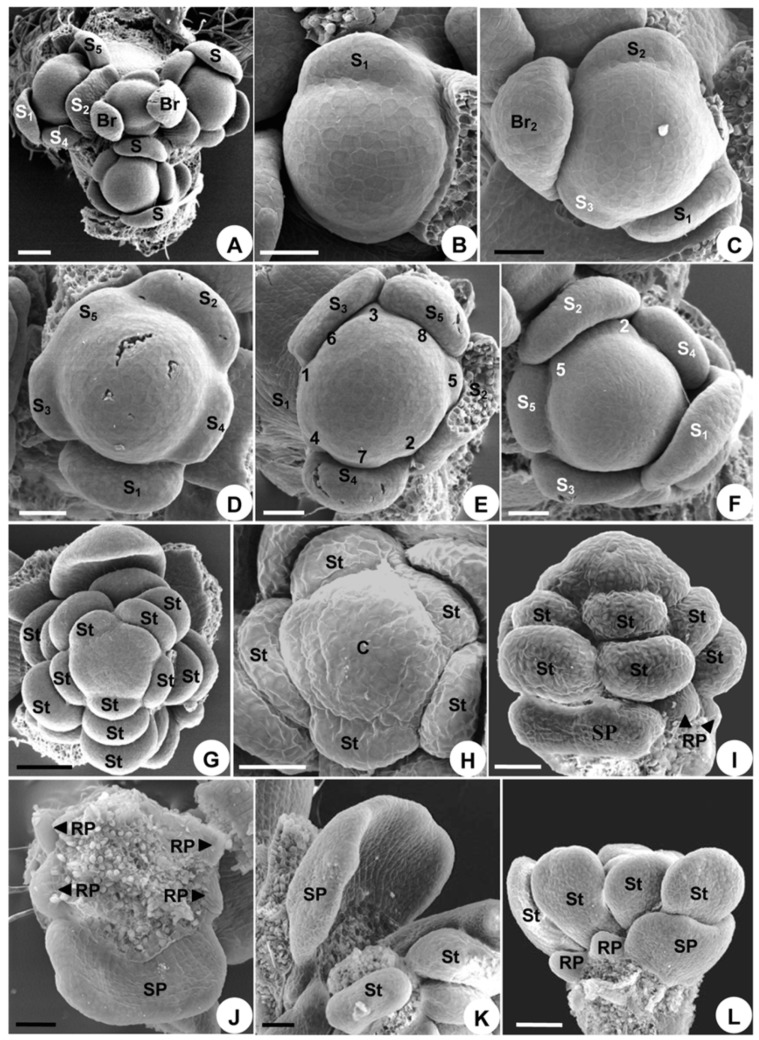
Floral organogenesis in *Consolida ajacis*. (**A**), initiation of floral apex, two bracteoles, and sepal primordia. (**B**), initiation of sepal 1. (**C**), initiation of sepals 1–3. (**D**), the outer pentamerous whorl of the five initiating sepals. (**E**), eight petal primordia initiate in a spiral order. (**F**), petals 2 and 5 are adaxial to sepal 2. (**G**), stamens arise, showing the orthostichies that consist of petals and stamens. (**H**), the carpel primordium initiates in the middle of the upper side of the floral primordium. (**I**), development of petals and stamens; two spurred petal primordia are confluent to form one, and the reduced petals cease growing shortly after initiation. (**J**), the confluent petal enlarges and the reduced petals show no further development. (**K**), the center of the petal depresses and arches inward. (**L**), side view of the spurred petal, reduced petal, and stamen, showing their size differences. Br, bracteole; C, carpel; RP, reduced petals; S, sepal; SP, spurred petal; St, stamen. Numbers indicate the initiation sequence. Scale bars: (**A**,**G**,**L**), 100 µm, (**B**–**F**,**H**–**K**), 50 µm.

The sequence of organ initiation resembles that observed in *A. taipeicum* and *D. giraldii,* with all floral organs occurring in a spiral pattern (except for the solitary carpel; Figure 8A–H). A unique feature of *C. ajacis* is that its single carpel emerges in the center of the floral apex after all other floral organs have been initiated (Figure 8H). Its petals and stamens are arranged in five regular orthostichies (Figure 8G).

#### 2.3.3. Organ Development

**Figure 9:**
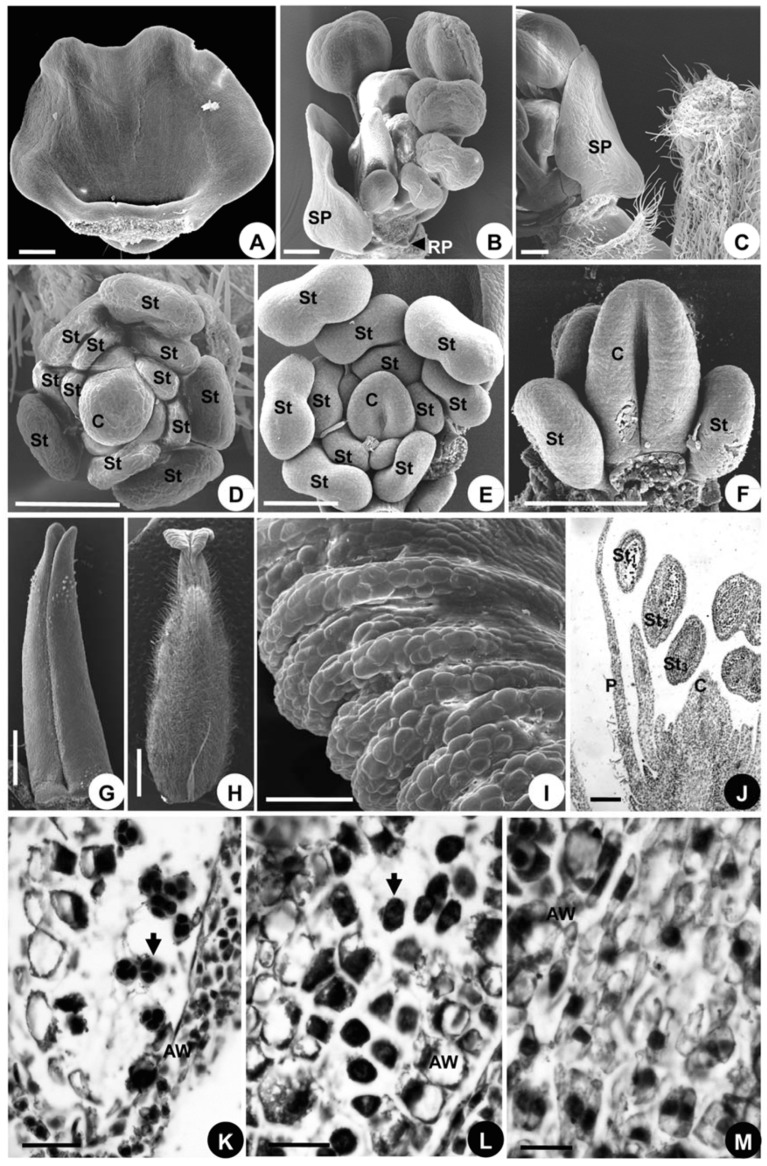
Floral organogenesis in *Consolida ajacis*. (**A**), the depressed petal becomes concave. (**B**), the spur on the petal appears as an outward growing pocket. (**C**), the outward growing pocket elongates to form a spur. (**D**–**F**), stamen and carpel development. (**D**), the centripetal development of stamens and the carpel begins to become concave. (**E**), the concave carpel appears to be horseshoe-shaped. (**F**), the cleft extends along the full length of the carpel. (**G**), the cleft closes except for the upper part. (**H**), the carpel differentiates into ovary, style, and stigma. (**I**), the surface of the stigma at anthesis. (**J**–**M**), microsporogenesis. (**J**), a longitudinal section of a flower showing three stamens. (**K**), the outermost stamen contains the microspores in the tetracyte stage (arrow). (**L**), the microspores are in the diad stage (arrow) in the middle stamen. (**M**), the innermost stamen contains microspores in the microsporocyte stage. AW, anther wall; C, carpel; P, petal; RP, reduced petals; SP, spurred petal; St, stamen. Numbers indicate the initiation sequence. Scale bars: (**A**–**G**,**I**–**M**), 200 µm; (**H**), 2 mm.

The development of the sepals also resembles that of *A. taipeicum* and *D. giraldii*, whereas the growth of the petals is quite different. Primordia 2 and 5 of the initiation sequence enlarge immediately after initiation. First, they become flat and confluent to form one, so that they appear to be a single petal (Figure 8I). This confluent petal shows a delayed growth when compared with the stamens that follow, but develops faster than the reduced petals (Figure 8I). The confluent petal soon grows to surround about half of the circumference of the floral meristem (Figure 8J). Thereafter, the petal enlarges and arches inward without forming any depression or stalk-like structure (Figure 8J), and then it continues to curve (Figure 8K,L). The center of the petals becomes depressed and arches inward, indicating the beginning of spur formation (Figure 9A). Later, outpocketing of the petal initiates (Figure 9B) and elongates to form the spur (Figure 9C). The other six reduced petals cease growing shortly after initiation (Figure 8I,J,L and Figure 9B). They develop initially as small scales (Figure 9B), but then during further development they completely disappear.

The stamens in the outer part of the androecium develop first, then the inner ones develop centripetally (Figure 8G and Figure 9B,D,E), and finally all stamens differentiate into filaments and anthers. This centripetal differentiation sequence is the same as that of *A. taipeicum* and *D. giraldii*.

The median, hemispherical, young carpel becomes concave and extends outward, rather than being erect (Figure 9D). Subsequently, this carpel elongates longitudinally to form a ventral suture, and thus appears horseshoe-shaped (Figure 9E). Later, the cleft reaches the upper side of the carpel, extending along its full length to the stigma (Figure 9F). Eventually, the cleft closes, but leaves the upper portion open (Figure 9G). After closure, and except for the upper portion, the carpel further differentiates into ovary, style, and stigma, with the stigma and style clearly separate. Trichomes begin to form on the ovary (Figure 9H). At anthesis, the carpel is covered with trichomes, except on the stigma (Figure 9H). The receptive surface of stigma expands downward and is covered with unicellular papillae (Figure 9I).

#### 2.3.4. Developmental Sequence of Microspores

Stamen maturation occurs in a centripetal direction, just as it does in *A. taipeicum* and *D. giraldii* (Figure 9J–M). Histological observations are made on the longitudinal section of a flower with three stamens (Figure 9J). The microsporogenesis of the outermost stamen contains the microspores in the tetracyte stage (Figure 9K), while the microspores are in the diad stage in the middle stamen (Figure 9L). The innermost stamen contains microspores in the microsporocyte stage (Figure 9M).

## 3. Discussion

### 3.1. Floral Ontogenesis Comparisons

The flowers of *A. taipeicum, D. giraldii*, and *C. ajacis* are all initiated in the axils of bracts. Their sepal primordia are crescent-shaped and truncate. Their petal, stamen, and carpel primordia are hemispherical, and all are smaller than those of the sepals. Petal and stamen primordia are similar in shape, size, internode length, and divergence angle. All floral organs are initiated in a spiral acropetal succession, whereas the carpel primordia arise almost simultaneously, generating a whorl-liked phyllotaxis of carpels. As a result, both spiral and whorl-liked arrangements coexist within a single flower. Ren et al. [48] showed a whorled initiation of tepal primordia in some flowers of *Anemone*. Kitazawa and Fujimoto [49] examined the perianth arrangement in *Anemone* and revealed that variation in organ arrangement is constrained in the spiral and whorled arrangements. The plastochron between the last sepal and the first petal is longer than that between the petals and stamens. The flowers first transform to a monosymmetrical structure when sepal 2 becomes the largest among other sepals. The second series, which consists of spurred, laminar, and reduced petals, shares a delayed developmental pattern. The two spurred petals form typical spurs and are concealed within sepal 2 (more or less similar spurs are also found in Ranunculaceae tribe Isopyreae [50]). In the extreme case, the two spurred petals of *C. ajacis* are completely fused, as described previously [4,17]. In summary, the three species have similar floral organogenetic and developmental patterns, with differences primarily occurring at the second series during the mid- to late-developmental stages. Furthermore, these ontogenetic observations largely agree with those previously reported in the tribe Delphinieae, showing many shared floral developmental characteristics [3,4,5,8,17,40,41,42,43,44,45,51]. Generally, the flowers of Delphinieae are radially symmetrical during organogeny and the monosymmetry is formed during the development of the second series [5,17,40,41,42,43,44,45].

Development of the second series in Ranunculales is rather complicated among basal eudicots [52], with Delphinieae being no exception. In Delphinieae, the second series of floral organs contains eight petal primordia, and their development is especially plastic and complex, with three interesting features emerging.

First, the delayed development of the second series can be interpreted as heterochrony, a temporal change in development. In *A. taipeicum*, the second series ceases to grow immediately after its initiation, with only the spurred petals continuing to grow. In *D. giraldii*, the eight petal primordia grow briefly after initiation, and then the reduced petals cease to grow. In *C. ajacis*, the delayed growth of the six reduced petals is more evident than that of the confluent petal, while the reduced petals disappear at anthesis. The delayed development of the petals and even the stamens on the outer whorl (i.e., the second series) has been widely documented in Ranunculaceae [4,8,13,34,46,53,54], the core eudicots [55], and even in other angiosperms [56]. Furthermore, the temporal and spatial correlations in delayed development, as seen in this study, within the second series have also been reported previously [5]. Heterochrony occurs through the delayed development of flowers at both intraspecific (i.e., developmental change in a species) and interspecific (i.e., developmental change between species) levels [57]. Heterochrony occurs presumably by the growth of multiple organ types on the second series. Heterochronic growth influences organ size, shape, and even the suppression of some organs or its parts. It is likely that there exists a relationship between the heterochronic growth of the second series and the type of floral organs produced. The systematic significance of heterochrony within the family is worthy of further study.

Second, the bulges or arches that form result in the petal spur structure. Petal primordia enlarge to form two bulges and a short stalk in *A. taipeicum*, a bulge without stalk in *D. giraldii*, or a sessile arch in *C. ajacis*. Previous studies have shown that in Ranunculaceae, the one or two bulges developing in the middle part of the adaxial side of the petal form either a spur or nectary [8,34,35,50,51]. Tamura [15] proposed that the stalk of the petal is of stylar origin. Erbar et al. [8] suggested that the ventral petal bulges represent the rudimentary adaxial pollen sacs of the stamen, while the stalk may represent the filament. Having two bulges and one stalk, as in *A. taipeicum*, likely represents a plesiomorphic state. The formation of an arch in *C. ajacis* is the result of the confluent petal being broad enough to make an arch and, subsequently, other curved structures, such as spurs.

Third, the second series of floral organs exhibits a trend of reduction that may parallel similar changes inferred during the evolutionary history of the petal. Spurred and reduced petals occur in all three species examined, whereas laminar petals occur in only *D. giraldii*. A complete disappearance of reduced petals during their developmental stage also occurs in *C. ajacis*. According to the process of floral elaboration during its course of evolution [56], the derived state is represented by *C. ajacis* because its two spurred petals are fused, the spur primordia at its early developmental stages is a sessile arch without the formation of bulge, and all reduced petals initiate, but disappear at anthesis. The formation of petaloid sepals is often correlated with a reduction in size or variability in the presence or absence of the petals in other angiosperm lineages, for example, Cunoniaceae, Oliniaceae, Polygonaceae, Saxifragaceae, and Thymelaeaceae [58], possibly because the petaloid sepals replace the petal as an optically attractive organ, allowing the petals to be reduced or lost altogether [2,58].

Ontogenetic studies of *Aconitum napellus* L. [8] and *Delphinium grandiflorum* L. (Figure 6 in the work of [5]) showed that primordia 2 and 5 of the initiation sequence are located in front of sepal 2. These results agree with our observations of *A. taipeicum*, *D. giraldii*, and *C. ajacis*, showing that it is primordia 2 and 5 that are located in front of sepal 2. The latter pair can be observed readily in *A. napellus* (Figure 3E in the work of [5]); *Callianthemum taipeicum* W. T. Wang and *Trollius farreri* Stapf (Figures 26, 27, 46, and 47 in the work of [34]); *Nigella damascena* and *Helleborus thibetanus* Franch. (Figures 2C,D, and 5B in the work of [35]); and *Ranunculus chinensis* Bunge, *Ceratocephala orthoceras* DC., and *Halerpestes cymbalaria* Greene (Figures 3c, 4e, 5d, 6c,d, and 7d in the work of [59]). In general, when there are five sepals, petals 2 and 5 of the initiation sequence are located in front of sepal 2, in either a clockwise or counterclockwise direction. However, when there are six sepals, such as in *Adonis sutchuenensis* Franch. (Figure 6 in the work of [34]) and *Ranunculus bungei* Steud. (Figure 5c in the work of [59]), primordia 1 and 4 of the initiation sequence are located in front of sepal 2.

### 3.2. Comparison of Tribe Delphinieae with Other Genera of Ranunculaceae

The tribe Delphinieae is considered to be a unique and advanced group in Ranunculaceae [25]. The genera of Delphinieae share organogenetic features with several other genera of Ranunculaceae, including *Anemone* L. [46,47]; *Caltha* L. and *Trollius* [33]; *Coptis* Salisb. [60]; *Helleborus* and *Nigella* [35]; and *Adonis* L., *Callianthemum* C.A.Mey., and *Trollius* [34]. The tribe Delphinieae also exhibits many unique characters, distinguishing them from other Ranunculaceae. As examples, the symmetry of the flowers varies among different floral organs, with both spiral and whorl-liked phyllotaxis coexisting in a single flower. Specifically, sepal 2 is the largest, but is not the first initiated, the petal forms a spur (a more or less similar spur is also found in Ranunculaceae tribe Isopyreae), and the two spurred petals of *C. ajacis* are fused into one from stalks to spur tips [4,17]. Partial fusion with only stalks fused, but two spur tips still free is observed in *Delphinium grandiflorum* L. [4,17], *D. macrocentron* Oliv. and *D. kingianum* Brühl ex Huth [61], and *Staphisagria macrosperma* Spach [17].

Besides the characters of the second series, the floral phyllotaxis also distinguishes the tribe Delphinieae. Here, both perianth and stamens show basically a spiral phyllotaxis, whereas the carpels show a whorl-liked phyllotaxis. In the family, both the spiral and whorled phyllotaxis can coexist at a specific level [13,34,62], but this coexistence rarely occurs in a single flower. Spiral phyllotaxis occurs widely in many taxa of Ranunculaceae [33,47,60,63], while a whorled phyllotaxis is found only in a group of closely related taxa, including *Aquilegia*, *Semiaquilegia*, and *Enemion* [50]. Delphinieae present low numbers of each type of floral organ, but possess both spiral and whorled floral phyllotaxis. Further sampling of species in the family is needed to illustrate a possible evolutionary relationship between floral organ number and phyllotaxis.

In many genera of Ranunculaceae, carpels generally initiate and arrange spirally [33,34,47,48,59]. When the number of carpels is few, such as in *Nigella* (5 carpels), *Helleborus thibetanus* (2 carpels) [35], *A. taipeicum* (5 carpels), and *D. giraldii* (3 carpels), then they generally initiate rapidly in a spiral sequence and are arranged as a whorl. These results suggest a correlation between the organ number and patterns of initiation and arrangement.

### 3.3. Comparison of the Tribe Delphinieae with Nigella

The tribe Delphinieae is closely allied with *Nigella* [7,11,26,27,28,29,30,64]. Developmental studies of *Nigella damascena* have identified floral characteristics shared with other members of Ranunculaceae [5,35]. As an example, the developmental pattern of the spurred petal in *Nigella* is highly similar to that of *Delphinium giraldii*, because both form an adaxial bulge at the base of each blade. Additionally, it has been reported that both *Delphinium* and *Nigella* form hairs and an abaxial bulge during petal developmental [5]. In this study, however, we did not observe this formation of hairs. Furthermore, the unique features of Delphinieae outlined above do not occur in *Nigella.*

Because of the variable numbers of petals in *N. damascena* (usually five to six, rarely up to eight), Zhao et al. [35] considered the relationship between this species and the tribe Delphinieae as unclear. In Delphinieae, eight petals in the second series is an invariant character [15], while eight petals are occasionally present in *Nigella*. It is hypothesized that Delphinieae shared an immediate common ancestor with *Nigella* [7,11,26,27,28,29,30,63].

### 3.4. Developmental Sequences of Stamens and Microspores

Various patterns of developmental sequences of stamens and microsporogenesis have been reported in Ranunculaceae, including centrifugal in *Aquilegia* [46,51]; centripetal in *Caltha* and *Trollius* [33], *Clematis* L. [48], *Thalictrum* L. and *Dichocarpum* W. T. Wang and Hsiao [36]; *Aconitum, Delphinium,* and *Consolida* (this study); and bidirectional in *Anemone* [47]. *Helleborus* is unique in having its stamen elongation and maturation sequences inverted [35]. The centripetal sequence is predominant in Ranunculaceae, as it appears to be in the tribe Delphinieae.

### 3.5. Floral Symmetry

The shift from actinomorphy towards zygomorphy is a late developmental event when it occurs in groups having predominantly actinomorphic flowers [40,65], such as Ranunculaceae. The flowers of Delphinieae are polysymmetrical at initiation and then transform to monosymmetry during the later developmental stages [5,40,41,42,43,44,45]. The zygomorphic phenotype can be more or less elaborate and possess various degrees of morphological differentiation [65]. The developmental processes of establishing a monosymmetrical structure are influenced by and strictly correlated with changes in the phenotypes of the floral organs.

Tucker [45] listed three conditions causing transition of floral symmetries: differential organ initiation; uneven enlargement of floral organs; and formation of modified structures (e.g., glands or spurs), with the latter two conditions being observed in the tribe Delphinieae. Previous studies have shown that the reduction of partial stamen whorls is correlated with the development of zygomorphy [12,65,66,67]. Structural monosymmetry may affect one or more floral whorls and requires modification, reduction, loss, or suppression of floral organs [67,68]. Our results show that the flower first manifests its monosymmetrical structure at the time when the calyx undergoes an unequal enlargement. This is also a common feature shared by many actinomorphic species of Ranunculaceae. Previous studies of floral organogenesis do not include the developmental process of sepals in forming monosymmetrical structures owing to the fact that the zygomorphy in Delphinieae is dependent on the structure of its corolla [5,8,40,41,42,43,44].

Our results showed that the flower obtains its monosymmetrical structure by modifying its existing floral traits. First, sepal 2, located abaxially or at the upper side of the flower, forms a helmet or spur. Second, petals 2 and 5, located in front of sepal 2, modify into spurred petals. Other petal primordia in the second series develop into reduced petals or reduce completely. The unequal growth of the perianth is also made possible by the monosymmetrical structure, which, for example, provides space along the symmetry plane for the upper primordia to develop into spurred sepal or petals.

The perianth symmetry is evident in Delphinieae. Carpel number is generally lower in Delphinieae than that of many other groups of Ranunculaceae. In Delphinieae, except for the solitary carpel in *C. ajacis*, the carpels initiated spirally are arranged in a whorl at the adult stage. In those members of Ranunculaceae having numerous carpels, for example, *Anemone* [47,48]; *Caltha* and *Trollius* [33]; *Adonis* and *Callianthemum* [34]; *Clematis* [48]; and *Ranunculus*, *Ceratocephala* Moench, *Halerpestes* Greene, and *Oxygraphis* Bunge [59], the carpel primordia initiate and arrange spirally. However, the relationships between floral monosymmetry and carpel number and their spatial arrangement are still unclear. Our observations suggest that the gynoecium may be affected by the reduction of the number of carpels and concomitant modification of the architectural structure owing to the spatial constraint of the flower.

A previous study focused on the same genera as our study, but of three different species (*Aconitum lasiocarpum, Delphinium elatum*, and *Consolida regalis*) to investigate the vascularization in the floral organs [69] based on the cross-sections of the flower buds and the hand-draw series of floral organs. Although both studies identified the spiral phyllotaxis in these plants, the scanning electron microscopy (SEM) images in our study evidently provided more detailed structures of the floral organs than the hand-drawn pictures, which unfortunately do not show the order of the floral organ initiation and development.

In summary, the monosymmetrical status of the flower is first established by the time sepal 2 becomes the largest. This feature of having the zygomorphic calyx is also found in many actinomorphic species of Ranunculaceae. This monosymmetry is increased by the unequal enlargement of the perianth, spur formation on the sepal, and the reduction and modification of the gynoecium. Previously, it was suggested that the integration of floral organs leads to the growth of the monosymmetrical structure [70]. A unique feature of *C. ajacis* is that its two petal primordia fuse into one, a phenomenon also observed in other Ranunculaceae, for example, *Delphinium*, *Aconitum*, and *Staphisagria* [4,17]. Further studies are needed to support and establish these observations of the growing pattern among the floral parts in monosymmetrical flowers.

## 4. Materials and Methods 

### 4.1. Species Examined

Floral organogenesis was examined in *Aconitum taipeicum* Hand.-Mazz., *Delphinium giraldii* Diels, and *Consolida ajacis* (L.) Schur. Flower buds of *A. taipeicum* and *D. giraldii* were collected in the Taibaishan Mountains (alt. 900–3500 m), Shaanxi, China. Flower buds of *C. ajacis* were obtained from a greenhouse at Northwest University, China. Voucher specimens were deposited in the Herbarium of the College of Life Sciences at Shaanxi Normal University (*A. taipeicum*, Bai Gen-Lu 2004011; *D. giraldii*, Bai Gen-Lu 2004002; *C. ajacis,* Chang Hong-Li 2010001).

### 4.2. Floral Organogenesis and Histological Studies

Flower buds were fixed in FAA solution (formalin/acetic acid/50% ethanol; 5:5:90 by volume). Prior to SEM, bracts, sepals, and stamens were removed from the flower buds using an Olympus SZX9 dissecting microscope with a cold light source. Dissections were dehydrated in 70% ethanol, subjected to an iso-amyl acetate series for 20 min, and then critical point dried using liquid CO_2_. Floral material was then mounted on aluminum stubs, coated with gold-palladium, and viewed with a Hitachi S-570 scanning electron microscope. Histological samples were dehydrated in an alcohol series, infiltrated with xylene, and embedded in paraffin wax. This embedded material was then sectioned at 8 µm thickness and stained with safranin and fast green. Photographs of mature flowers were taken with a Nikon Coolpix 990 digital camera.

## 5. Conclusions

In this study, we used scanning electron microscopy and histological methods to study three species in the tribe Delphinieae of Ranunculaceae (*Aconitum taipeicum*, *Delphinium giraldii*, and *Consolida ajacis*). The results showed four unique features in the members of Delphinieae that are different from their polysymmetrical relatives in their development of the spiral phyllotaxis of their perianth, stamens, and whorled carpels, the sepal 2, the petals 2 and 5, and the monosymmetry of the first flower. Major ontological differences among the three species included the timing of development of the second series; the fusion of two petals into a single one in *C. ajacis*; and, during early developmental stages, the two young spurred petals giving rise to a stalk and two bulges in *A. taipeicum*, a single bulge in *D. giraldii*, or an arch blade in *C. ajacis*. These results demonstrated that the unequal growth of the perianth, together with the reduction and the rearrangement of the carpels, are critical in inducing the symmetrical transformation of the flowers. Given the unique type of symmetrical pattern of the floral organs presented in these species, it is important to reveal the functions of floral development related genes (e.g., MADS-box and TCP transcription factor genes) and to elucidate the molecular and genetic mechanisms underlying the floral organ identity and initiation in these model systems. Our study provides the necessary morphological and developmental characterizations of the florogenesis in these plants for the potential molecular and genetic investigations.

## Figures and Tables

**Table 1:** Comparison of floral organogenesis in five members of tribe Delphinieae and *Nigella*. Superscripts ^1^, ^2^, and, ^3^ indicate the data collected from the works of [5], [35], and [8], respectively. N/A indicates data not found or absent.

Floral Organ and Trait	*Aconitum taipeicum*	*Aconitum napellus* ^1^	*Delphinium giraldii*	*Delphinium grandiflorum* ^1^	*Consolida ajacis*	*Nigella damascena* ^2^
Sepal						
Initiation pattern	spiral	spiral	spiral	spiral	spiral	spiral
No. of sepals	5	5	5	5	5	5
The largest sepal	sepal 2	N/A	sepal 2	N/A	sepal 2	sepal 1
Appendant on sepal 2	helmet	helmet	spur	spur	spur	N/A
Spurred petal						
Initiation pattern	spiral	spiral	spiral	spiral	spiral	spiral
No. of spurred petals	2	2	2	2	1	5–8
Status at anthesis	separate	separate	separate	separate	connate	separate
Development of spur						
Primordia growing into spurred petals	petals 2 and 5	petals 2 and 5	petals 2 and 5	petals 1 and 4	petals 2 and 5	N/A
Stalk	present	present	absent	absent	absent	present
No. of bulges	2	N/A	1	N/A	0	1^1^
Depression	present	present	present	N/A	absent	present
Reduced petal						
No. of reduced petals	6	6	4	4	6	0
Status at anthesis	present	present	present	present	absent	N/A
Plate petal (No.)	absent	absent	Present (2)	Present (2)	absent	absent
Stamen						
Initiation pattern	spiral	spiral	spiral	spiral	spiral	spiral
No. of anthers	35–45	50^3^	18–30	N/A	13–20	30–45
Development sequence	centripetal	centripetal	centripetal	centripetal	centripetal	centripetal
Anther maturation pattern	centripetal	N/A	centripetal	N/A	centripetal	centripetal
Carpel						
Initiation pattern	spiral	spiral	spiral	spiral	spiral	spiral
No. of carpels	5	3	3	3	1	3–5
Arrangement	whorled	whorled	whorled	whorled	N/A	whorled
Floral organ arrangement	parastichy	parastichy	parastichy	parastichy	orthostichy	parastichy
